# Task-Oriented Training for Rehabilitation in Multiple Sclerosis in a Non-Hospital Setting: A Protocol for a Randomized Controlled Trial

**DOI:** 10.3390/healthcare14091163

**Published:** 2026-04-27

**Authors:** Alba Navas-Otero, Mirella Villegas-López, Jessie Jambón-Folea, Susana Varón-Jiménez, Irene Cabrera-Martos, Araceli Ortiz-Rubio, María del Carmen Rodríguez-Martínez

**Affiliations:** 1 Department of Physical Therapy, Faculty of Health Sciences, University of Granada, 18071 Granada, Spain; 2 Department of Physical Therapy, Faculty of Health Sciences, University of Málaga, 29071 Málaga, Spain

**Keywords:** fatigue, functionality, multiple sclerosis, rehabilitation, task-oriented training

## Abstract

**Highlights:**

**What are the main findings?**

- This approach supports motor recovery while also facilitates the transfer of learned skills to meaningful daily life activities.
- A focus on functional performance allows rehabilitation to be directly aligned with MS patients’ daily life needs and priorities.

**What are the implications of the main findings?**

- Clinicians should address not only physical impairments but also how MS affects daily activities and functionality.

**Abstract:**

**Objective**: The aim of this study will be to evaluate the effectiveness of a task-oriented training program in improving functional performance and health outcomes in patients with MS. **Methods**: A pilot randomized clinical trial will be conducted according to SPIRIT guidelines. Participants will be randomly assigned to the experimental group or the control group. Assessment and treatment will take place at patient association facilities or research center. Participants diagnosed with MS by a neurologist and meeting the inclusion criteria will be invited to participate voluntarily. The experimental group will undergo an 8-week TOT intervention, twice weekly, 45 min. The control group will maintain usual care and be given a fatigue management pamphlet. The main variable in this study will be the Canadian Occupational Performance Measure. The level of fatigue will be assessed with the Modified Impact Fatigue Scale and the Fatigue Severity Scale, upper limb strength using the Arm Curl Test, hand and pinch dynamometer, motor speed with the Finger Tapping Test, manual dexterity with the Nine Hole Peg Test, Purdue Pegboard Test and the Coin Rotation Test. Satisfaction and adherence with the intervention will be recorded with the Sport Injury Rehabilitation Adherence Scale. **Results**: The results will be published as a peer-reviewed article. **Conclusions**: The present protocol aims to fill a relevant gap in the literature, offering a structured intervention based on task-oriented training principles, specifically tailored to the functional and occupational needs of people with MS.

## 1. Introduction

Multiple sclerosis (MS) is a chronic, progressive, and demyelinating disease of the central nervous system. It is considered one of the most common non-traumatic disabling diseases that affects young adults [1]. This disease causes sensorimotor, visual, cognitive and autonomous dysfunctions [2]. Of particular relevance, fatigue stands out as one of the most prevalent and disabling symptoms among MS patients and causes interference with patients’ daily functioning [3]. Upper extremity function, manual dexterity, and walking ability are also recognized as key predictors of perceived difficulties in performing daily activities among individuals with MS [4]. It significantly affects health and leads to a wide range of impairments in body functions, limitations in activities, and restrictions in participation [5]. As a result, the performance of daily life activities may be considerably compromised, thereby increasing the need for care and support for these patients.

In this context, task-oriented training (TOT) interventions have emerged from the movement science and motor skill learning literature [6]. This perspective of rehabilitation focuses on improving performance in functional tasks through mass goal-directed practice and repetition of activities that are of high intensity and meaningful to the patient. Bayona et al. [7] reported the importance of TOT interventions, noting that the most effective way to relearn a task is to train specifically for that task. It has been developed within the literature of motor control and learning, with increased evidence of neural plastic changes as a result of this training being shown [8]. Previous studies have underscored that TOT seeks to enhance cortical reorganization, remediation and the reacquisition of functional skills by encouraging active problem-solving, while systematically varying environmental conditions, feedback and task difficulty to improve performance [9].

The efficacy of TOT interventions has been previously demonstrated in some studies showing improvements in different health outcomes, such as upper limb function [10], manual dexterity [11], and walking endurance [12], among MS patients. However, authors have highlighted the need for additional research to strengthen the current body of evidence. While TOT has been widely implemented and extensively studied in stroke patients, its application in MS remains in the early stages. Therefore, this study aims to evaluate the effectiveness of a TOT program in improving functional performance and health outcomes in patients with MS.

## 2. Materials and Methods

### 2.1. Study Design

A pilot randomized clinical trial will be conducted in accordance with the guidelines of Standard Protocol Items: Recommendations for Interventional Trials (SPIRIT) [13]. The study is registered at clinicaltrials.gov with the code NCT07106255.

### 2.2. Ethical Aspects

This study will comply with and respect the principles established in the Declaration of Helsinki of 1964 and its subsequent amendment in 2013 (World Medical Association, 2013) [14], which states that the purpose of biomedical research involving human subjects must focus on “improving diagnostic, therapeutic, and prophylactic methods, as well as the understanding of the etiology and pathogenesis of diseases.” Similarly, the study will adhere to the International Ethical Guidelines for Biomedical Research Involving Human Subjects of 1982. These guidelines emphasize the protection of the most vulnerable communities to ensure that the rights of every age group or individuals with specific pathologies are respected. The study will also comply with the provisions of Organic Law 3/2018 of 5 December on the Protection of Personal Data (LOPD), ensuring the confidentiality of the data of the participants included in the study. A certificate from the Regional Ethics Committee (Andalusian Biomedical Research Ethics Portal) was obtained with code 2036-N-23.

### 2.3. Participants

Participants with MS will be invited to voluntarily participate in different patient associations. They will be informed about the objective and development of the study and sign an informed consent prior to their inclusion. The inclusion criteria for the MS group will include the following: (1) Diagnosis of Multiple Sclerosis by a neurologist according to the 2024 revisions of the McDonald Criteria [15]. These criteria require evidence of dissemination in space, defined by typical lesions in at least two of five anatomical locations within the central nervous system (CNS) (periventricular, cortical or juxtacortical, infratentorial, spinal cord, and optic nerve). Diagnosis must be confirmed by the presence of dissemination in time or evidence of intrathecal antibody production (demonstrated by cerebrospinal fluid-restricted oligoclonal bands or a positive kappa free-light chain index). Alternatively, MS may be diagnosed if patients show typical clinical presentations involving four or more typical CNS anatomical locations, or through the identification of specific MRI biomarkers such as the central vein sign or paramagnetic rim lesions in required clinical and radiological contexts. (2) Participants must be aged over 18 years old. (3) Participants must accept and sign the informed consent. Participants will be excluded if they present (1) a cognitive level or condition that did not allow for completion of the evaluations; (2) an exacerbation of MS during the previous 3 months; (3) the presence of interfering musculoskeletal, neurological, orthopedic, and/or rheumatic disorders affecting hand and/or finger mobility, including previous trauma or fracture of the upper extremity. Recruitment will remain open until July 2026.

### 2.4. Procedures and Measures

**Figure 1:**
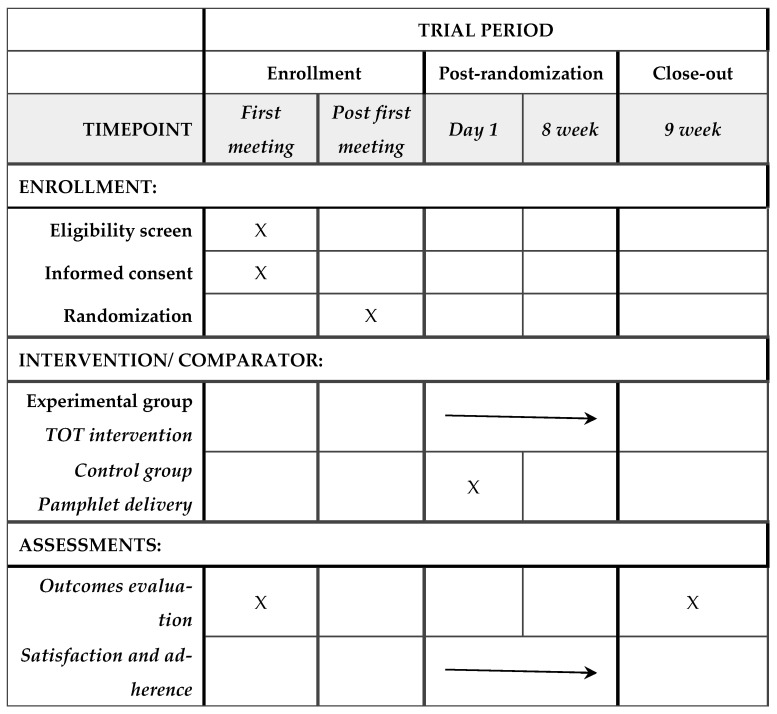
Participant timeline: Schedule of enrollment, interventions, and assessments.

The evaluation and the intervention will be conducted either in the facilities provided by the MS associations participating in this study or in the facilities of the research center. The specific location will be selected according to the participant’s preference, to facilitate accessibility and adherence to the intervention. The participant timeline, including the schedule of enrollment, interventions, and assessments, can be found in Figure 1.

#### 2.4.1. Allocation

After eligibility assessment, participants will be randomly assigned to either the experimental group or the control group. Allocation will be determined by using a computer-generated random number list with the software www.random.org.

#### 2.4.2. Experimental Group

Participants included in the experimental group will undergo an 8-week TOT intervention, administered twice weekly, with each session lasting 45 min. TOT “*is an effective rehabilitation intervention that focuses on the use of client-centered, repetitive practice of activities that are of high intensity and meaningful to the client*” [9]. The TOT intervention will be designed based on a previous study [16] that established guidelines for its implementation in clinical practice. Within this framework, the intervention will comprise sessions involving meaningful and repetitive activities tailored to both the patient and their specific context. The tasks included in the intervention sessions will be chosen by each participant based on the occupations identified through the Canadian Occupational Performance Measure (COPM) [17] tool during the initial assessment. Through the COPM semi-structured interview, participants will identify daily activities they find difficult to perform and rate their importance on a 10-point scale. The three activities rated as most important by the participant will be selected as the primary treatment goals. This client-centered selection process directly dictates the content of the intervention, ensuring that all sessions will be tailored to practice and improve the specific daily activities chosen by the participant. The tasks derived from the three selected occupations will be randomly organized and focused on their complete reconstruction. To support the intervention, a range of custom-designed materials will be employed, alongside therapeutic tools and materials such as therapeutic putty, TheraBand, and other tools commonly used in physical rehabilitation interventions. Throughout the intervention, participants will receive positive reinforcement that will fade over time to prevent unnecessary dependency. To ensure optimal therapeutic dosing and standardize difficulty progression across participants, task intensity will be monitored using the Borg Rating of Perceived Exertion (RPE, 6–20) scale [18]. This tool consists of a scale from 0 to 20 levels of perception, with the number 6 being ‘very, very light’, while a value of 20 is considered as ‘very, very hard’. The complexity of the selected task will systematically be increased only when the participant consistently reports a perceived exertion level of 11 (“Fairly light”) while performing the task. Conversely, the complexity will decrease if the participant reports a level of 17 (“Very hard”). The use of this standardized threshold ensures that all participants remain in an optimal challenge zone for motor learning and neuroplasticity, dictating the moment for task progression regardless of the specific nature of the practiced occupation [19,20]. Once the participant achieves and maintains a perceived exertion level of 11 on the Borg scale, the therapist implements systematic modifications to progress the difficulty or introduce contextual interference. To standardize these modifications across the entire sample, regardless of the selected occupation, therapists will use the task-adjustment parameters described in Supplementary Materials.

#### 2.4.3. Control Group

Participants included in the control group will continue with their usual treatment and will receive a pamphlet containing information on fatigue management. The selection of usual care as a comparator will be based on the pragmatic nature of the research question, which seeks to evaluate the effectiveness of the intervention against existing clinical practices. By utilizing this design, we aim to measure the incremental benefit of the intervention relative to the outcomes of patients receiving individualized care in a real-world clinical environment [21].

#### 2.4.4. Descriptive Measures

An independent researcher, blinded to the patients’ group allocation, will collect all data at baseline and after the intervention. To characterize the clinical profile of the participants included in the study, the Expanded Disability Status Scale (EDSS) [22] and the ‘symptoMScreen’ [23] will be used. The EDSS is a widely used method for quantifying disability in individuals with multiple sclerosis. It ranges from 0 (normal neurological function) to 10 (death due to MS), based on neurological examination and the assessment of ambulation and functional systems. The ‘symptoMScreen’ tool consists of 12 domains, each rated on a 7-point Likert scale ranging from 0 (not affected at all) to 6 (total limitation/unable to perform most daily activities). In addition, sociodemographic and anthropometric characteristics will also be collected, including age, sex, and type of MS.

#### 2.4.5. Outcome Measures

The primary outcome in this study will be the Canadian Occupational Performance Measure (COPM) [17]. This tool measures the perception of problems related to the overall quality of performance, development and satisfaction with particular occupations. This is a standardized instrument that was previously validated [17,24] and used [25] in MS patients. The COPM will be used under a formal license that will be obtained through official channels to ensure full compliance with copyright and intellectual property regulations.

The secondary outcomes include fatigue level, upper limb strength, motor speed and manual dexterity. The level of fatigue will be assessed with the Modified Impact Fatigue Scale (MFIS) [26] and the Fatigue Severity Scale (FSS) [27]. The MFIS consists of items from which a total score is obtained and for each subscale. Scores range from 0 to 36, with higher scores indicating a greater impact of fatigue on activities. The FSS consists of nine items that are rated on a scale of 1 to 7. A higher score indicates a higher agreement with the statement presented in each item. This scale is recommended for use in clinical practice and research in MS patients [26,28].

Upper limb strength will be assessed using the Arm Curl Test (ACT) [29], a hand dynamometer, and a pinch dynamometer. These tests have previously been used in MS research [30,31]. The ACT consists of performing as many arm curls as possible in 30 s while sitting in a chair. The dynamometer and hand dynamometer measure grip and pinch strength respectively. Motor speed will be assessed with the Finger Tapping Test (FTT) [32]. In this test, the number of fingers taps the participant can perform in three attempts of 20 s each is counted. Manual dexterity will be assessed using the Nine Hole Peg Test (9HPT) [33], Purdue Pegboard Test [34] and the Coin Rotation Test (CRT) [35]. All these tests have previously been used and validated among MS patients [33,36,37]. The 9HPT requires the participant to place and then remove nine pegs, one at a time, into nine holes on a board as quickly as possible. The test is performed separately with each hand, and the time taken to complete the task is recorded. Shorter completion times indicate better manual dexterity. The Purdue Pegboard Test involves placing as many pins as possible into holes on a board. It includes subtests for the dominant and non-dominant hand, both hands simultaneously, and an assembly task. Higher scores reflect better dexterity. CRT assesses the coordination and speed of the fingers by rotating a coin clockwise with three fingers of the hand (thumb, index and middle finger) as fast as possible.

Satisfaction and adherence with the intervention will be recorded with the Sport Injury Rehabilitation Adherence Scale (SIRAS) [38]. This test consists of three clinician-rated items scored on a 5-point Likert scale (1–5): (1) the intensity of effort with which the patient completes the rehabilitation exercises (from minimum effort to maximum effort), (2) the extent to which the patient follows instructions and advice (from never to always), and (3) the patient’s receptivity to changes in the rehabilitation program (from very unreceptive to very receptive). In this context, a score of 1 reflects minimal engagement, whereas a score of 5 indicates optimal adherence. Although originally developed within a sports medicine context, the SIRAS evaluates three universal behavioral components of physical rehabilitation: the patient’s intensity of effort, adherence to clinician instructions, and receptivity to therapeutic changes. Because these observational metrics assess the active, behavioral aspects of patient engagement during supervised sessions—rather than being dependent on the physical etiology of the condition—the SIRAS provides a valid, reliable, and highly transferable measure of session adherence among MS patients.

#### 2.4.6. Sample Size Calculation

The sample size has been calculated using the software G*Power 3.1.9.7 (3.1.9.7v; Statistical Power Analyses for Windows, Universität Düsseldorf, Germany). The calculation was based on the COMP tool. According to a previous study [39], assuming an effect size of 0.7 for the COMP tool, an alpha value (α) of 0.05, a power (1 − β) of 0.80, and a 1:1 randomization ratio, a total of 52 patients will be needed. Assuming a 10% dropout rate, a total of 58 participants should be included in this study (29 participants in the experimental group and 29 participants in the control group).

#### 2.4.7. Statistical Analysis

Data analysis will be performed using the Statistical Package for the Social Sciences version 24 (SPSS v24). A fully licensed version of SPSS is available through the university tp which the corresponding author of this manuscript belongs.

Descriptive statistics will be used to summarize the sample characteristics. Qualitative variables will be presented as percentages (%), while quantitative variables will be reported as mean ± standard deviation or as median and interquartile ranges (IQR).

The statistical distribution of the data will initially be assessed using the Shapiro–Wilk test. To ensure baseline balance, demographic characteristics and initial assessments will be compared between groups using independent samples *t*-tests for normally distributed data, the Mann–Whitney U test for non-normally distributed variables, and Chi-square tests for categorical data.

The primary analysis of the intervention’s effect will be conducted using a two-way mixed-design ANOVA, with ‘Group’ (Experimental vs. Control) as the between-subjects factor and ‘Time’ (Pre- vs. Post-intervention) as the within-subjects factor. The Group × Time interaction will be the primary effect of interest to determine efficacy. In the event of significant interactions, post hoc pairwise comparisons with Bonferroni correction will be performed.

Two-sided 95% confidence intervals will be calculated, and statistical significance will be set at *p* ≤ 0.05. No subgroup or exploratory analyses are planned for this study to maintain adequate statistical power.

## 3. Results

The results will be published as a peer-reviewed article. We will summarize the results in three tables. The first table will outline the descriptive characteristics of the sample. The second table will compare the primary outcomes between groups at baseline. The third table will present post-intervention outcomes, detailing both intra-group and between-group changes, with effect sizes calculated using Cohen’s d. Additionally, the article will include a figure illustrating the flow diagram.

## 4. Discussion

This protocol for a randomized controlled trial is based on the growing evidence supporting the use of TOT as an effective intervention in neurological rehabilitation. Due to the multidimensional nature of MS symptoms, the selection and design of effective interventions represent a major challenge.

In this context, TOT is a promising approach not only for promoting motor recovery but also for generalizing learned skills into meaningful daily life performance [40]. This makes it a strategy aligned with the fundamental values of occupational therapy and rehabilitation professionals, such as the active participation of the patient in the choice of goals and in the planning of rehabilitation [41].

TOT focuses on enhancing motor learning by engaging sensory functions through repetitive movement, active participation and specific task training, which are adjusted based on sensory feedback and perception [16,42]. This approach can encourage motor recovery by developing excitability in the primary motor area [43] and by inducing central nervous system reorganization [44,45]. It is important to consider that repetitive training alone may not be sufficient to induce changes in cortical representation unless it is associated with learning specific skills, in line with a model of learning-dependent neural plasticity [46]. It has been shown to positively influence the ability to perform functional tasks, contributing to overall motor function improvement in this regard, which ultimately enhances functional performance.

This promotion of functional recovery is rooted in the fundamental principles of experience-dependent neural plasticity. As established in a previous study [47], the principle of specificity dictates that the nature of the training experience determines the nature of the plasticity. Specifically, significant changes in neural connectivity and motor map expansion require skill acquisition rather than mere motor use. Moreover, our study emphasizes ‘meaningful’ activities to align with the principle of salience, which posits that an experience must be sufficiently important or relevant to the person to drive plastic changes. Consequently, by combining high-dose repetition with goal-directed tasks, the proposed TOT intervention utilizes the brain’s intrinsic mechanisms for learning and relearning.

Previous systematics reviews exploring the effects of TOT have concluded that it is a safe and feasible intervention [48]. Although this approach clearly aligns with the theoretical and philosophical principles of occupational science and client-centered interventions, its use in clinical practice is still emerging. This is because rehabilitation is commonly based on accepted practice or custom. Moreover, the existing literature around MS rehabilitation predominantly focuses on impairments in body functions, frequently neglecting activity limitations and participation restrictions, which are central concerns in these patients [49]. This approach draws heavily on enduring professional rehabilitation principles that prioritize the active participation of patients in establishing goals and shaping their rehabilitation processes.

In contrast to conventional TOT interventions, the design of this study prioritizes treatment personalization using the COPM [17]. This methodological choice ensures that interventions are highly meaningful to each participant. By aligning the rehabilitation goals with the actual functional needs and priorities of the participants, the transfer of learned skills to real-world daily functioning becomes more achievable. To ensure reliability and reproducibility, regardless of the specific tasks selected, the training intensity remains comparable thanks to the use of the Borg Scale [18]. By using this, the protocol guarantees that every participant operates within an optimal challenge zone for motor learning and neuroplasticity. Moreover, the study utilizes task-adjustment parameters, providing therapists with a systematic structure to modify task demands.

A notable limitation of this design is that difficulty progression relies on the patient’s subjective perception of effort. While the Borg scale is a validated tool in MS populations [19], the fact that task progression is based on self-reported exertion rather than objective kinematic or performance measures may introduce variability in the delivered dose.

## 5. Conclusions

Accordingly, the present protocol aims to fill a relevant gap in the literature, offering a structured intervention based on task-oriented training principles, specifically tailored to the functional and occupational needs of people with MS. It is hoped that the results of this study will contribute to strengthening the evidence around this strategy, promoting a clinical practice more focused on occupational performance and the meaningful participation of patients.

## Data Availability

No new data were created or analyzed in this study.

## Supplementary Materials

The following supporting information can be downloaded at: https://www.mdpi.com/article/10.3390/healthcare14091163/s1.

## Abbreviations

The following abbreviations are used in this manuscript:

ACT	Arm Curl Test
COMP	Canadian Occupational Performance Measure
CRT	Coin Rotation Test
CONSORT	Consolidated Standards of Reporting Trials
EDSS	Expanded Disability Status Scale
FTT	Finger Tapping Test
MFIS	Modified Impact Fatigue Scale
MS	Multiple Sclerosis
9HPT	Nine Hole Peg Test
LOPD	Organic Law 3/2018 of December 5 on the Protection of Personal Data
SIRAS	Sport Injury Rehabilitation Adherence Scale
SPIRIT	Standard Protocol Items: Recommendations for Interventional Trials
TOT	Task-oriented training
