# Supplementary material for: Task-Oriented Training for Rehabilitation in Multiple Sclerosis in a Non-Hospital Setting: A Protocol for a Randomized Controlled Trial
Source: Healthcare (Basel). 2026 Apr 27;14(9):1163. doi: 10.3390/healthcare14091163 (PMC13163390; doi:10.3390/healthcare14091163)
Supplement: Supplementary file 1 [file healthcare-14-01163-s001.zip › healthcare-4247423-supplementary.pdf]

Supplementary Materials. Task-adjustment parameters for the experimental group

- **Changes in the workspace:** Altering the placement of targets and the spatial arrangement of objects relative to the person (e.g., increasing reaching distance or changing the plane of movement).
- **Changes in object characteristics:** modification of the weight, size, texture, shape, or material of the objects manipulated during the task.
- **Changes in patient positioning:** Progressing the biomechanical and balance demands by transitioning the execution of the task from a sitting to a standing position.
- **Variability within the task:** Introducing random practice by continuously alternating object characteristics or environmental conditions in subsequent repetitions of the same task, aiming to prevent motor automation and foster motor adaptability.
- **Part practice:** The task will be divided into its underlying skill components. These parts are practiced in isolation and then immediately combined and reassembled, thereby fulfilling the ultimate goal of the TOT approach to achieve the reconstruction and performance of the whole task.
